# Evaluation of the effects of wood-sourced biochar as a feedlot pen surface amendment on manure nutrient capture

**DOI:** 10.1093/tas/txac127

**Published:** 2022-09-10

**Authors:** Jessica L Sperber, Galen E Erickson, Andrea K Watson

**Affiliations:** Department of Animal Science, University of Nebraska, Lincoln, NE 68583-0908, USA; Department of Animal Science, University of Nebraska, Lincoln, NE 68583-0908, USA; Department of Animal Science, University of Nebraska, Lincoln, NE 68583-0908, USA

**Keywords:** biochar, cattle, feedlot manure, mass balance

## Abstract

Feedstuffs utilized in U.S. feedlot finishing rations incorporate high concentrations of N and P, with less than 15% of fed N and P retained by the animal. The remaining N and P are excreted in the manure, where the opportunity for manure N loss via ammonia (NH3) volatilization from the feedlot pen surface is a risk to the environment and lowers the value of manure as a fertilizer. Two nutrient mass balance experiments were conducted during the winter and summer seasons to evaluate the effects of spreading unprocessed Eastern red cedar biochar onto the feedlot pen surface on manure nutrient capture and cattle performance. A 186-d feedlot finishing experiment was conducted from December to June (WINTER) and a subsequent 153-d finishing experiment was conducted from June to November (SUMMER). The WINTER experiment evaluated three treatments (5 pens per treatment; 10 steers per pen), including biochar spread on pen surface during the feeding period (1.40 kg biochar/m^2^; 17.6 m^2^/steer soil surface of the pen), hydrated lime spread on pen surface at end of feeding period (1.75 kg/m^2^) and control (no treatment applied). The SUMMER experiment evaluated biochar treatment (1.40 kg biochar/m^2^; 5 pens per treatment; 8 steers per pen; and 22 m^2^/steer soil surface of the pen) against control. There were no differences in N and P intake, retention, or excretion (*P* ≥ 0.38) between WINTER treatments. Steer performance (*P* ≥ 0.10) and carcass characteristics (*P* ≥ 0.50) were not impacted by pen treatment in WINTER. Nitrogen and P intake and excretion (*P* ≥ 0.35) were not different between treatments in SUMMER and retention of N and P was significantly greater for the biochar treatment (*P* ≤0.04) due to greater ADG (*P* = 0.05). There was no difference in DMI (*P* = 0.48) in SUMMER, steers on biochar pen treatment had heavier HCW (*P* = 0.05) and greater ADG, resulting in a tendency for greater feed efficiency (*P* = 0.08). In both experiments, biochar addition to the pen surface tended (*P* = 0.07) to increase manure N as a percent of manure DM, but this increase in N concentration did not impact kg of N removed from the feedlot pens (*P* ≥ 0.15) or N losses (*P* ≥ 0.68). The addition of red cedar biochar to the feedlot pen surface did not increase manure nutrient capture of N or P and did not reduce N losses associated with soil-based feedlot pens.

## INTRODUCTION

Typical beef feedlot finishing diets in the U.S. combine high inclusions of concentrate (grains) with a variety of byproducts, crop residues, and forages that incorporate high quantities of N, P, and soluble salts. Of total N offered in the diet, beef cattle retain approximately 12% of fed N and 15% of fed P (NASEM, 2016), with the remainder excreted onto the feedlot pen surface. Once excreted onto the pen surface, manure nutrients can be 1) volatilized as ammonia (NH_3_) or nitrous oxide (N_2_O); 2) volatilized as carbon dioxide (CO_2_) or methane (CH_4_); 3) lost as precipitative runoff and captured in a runoff retention pond; or 4) removed in manure during pen cleaning. The opportunity for manure N loss from open dirt feedlot pens is a risk to the environment, public health, and poses a potential economic loss in the value of manure as a fertilizer. The environmental risks include water quality concerns from the deposition of N from manure fertilizer application contributing to eutrophication, air quality degradation due to NH_3_ volatilization, and the potential for nitrous oxide (N_2_O) formation and its implications as a GHG on climate change (USDA Agricultural Air Quality Task Force, 2014; USEPA, 2004).

Immediately following excretion of urine and feces from the animal, NH_3_ formation and volatilization occur rapidly due to abundant urease activity in the feces and soil (Bussink and Oenema, 1998), with manure N losses via volatilization ranging from 43% to 64% of fed N (Homolka et al., 2021). Seasonal differences in manure N loss via NH_3_ volatilization occur due to the impacts of temperature and moisture conditions on feedlot pen surface microbial activity and the speed of chemical reactions with greater losses occurring during summer feeding periods (Gilbertson et al., 1971; Homolka et al., 2021; Hristov et al., 2011; Kissinger et al., 2007). Various feedlot management strategies, such as sawdust application (Lory et al., 2002) and increased dietary bran inclusion (Adams et al., 2004), have shown that as OM content is increased on the pen surface, greater N is retained in the manure and N losses via NH_3_ volatilization are reduced.

One proposed method of improving manure nutrient capture of N and P is to apply biochar to the feedlot pen surface. Biochar is produced by burning OM (typically forest industry byproducts) at high temperatures in the absence of oxygen (Hansen et al., 2012) resulting in an end-product that is high in C content. When biochar is utilized as a soil amendment, improvements in crop yields and soil fertility (Atkinson, Fitzgerald and Hipps, 2010; Ding et al., 2016) and reductions in emissions of N_2_O and CH_4_ from crop fields have been observed (Cayuela et al., 2014; Karhu et al., 2011). The addition of biochar to various livestock wastes has resulted in reductions in N_2_O (Agyarko-Mintah et al., 2017; Brennan et al., 2015; Li et al., 2016), NH_3_ (Brennan et al., 2015; Chen et al., 2021; Maurer et al., 2017), CO_2_ (Brennan et al., 2015), and P (Streubel et al., 2012) losses, due to the high surface area, porosity, and cation exchange capacity of biochar (Agyarko-Mintah et al., 2017; Kammann et al., 2015; Streubel et al., 2012). Agyarko-Mintah et al. (2017) reported a 65% to 75% reduction in N_2_O emissions (mg/kg of compost-biochar mixture) when biochar made from litter and green waste was added to a compost mixture of poultry litter and straw at 10% of compost dry matter. Brennan et al. (2015) reported biochar made from wood shavings reduced emissions of NH_3_ (77%), N_2_O (63%), and CO_2_ (84%) from dairy cattle manure slurry when applied at a rate of 3.96 m^3^/ha. However, these benefits have not been demonstrated in open lot feedlot pens, which produce manure with very different physical characteristics than swine, dairy, or poultry manure systems. In addition to reducing nutrient losses from livestock waste, adding biochar to the feedlot pen surface may also improve pen surface conditions by reducing moisture on the pen surface (Maharjan and Wilke, 2021).

The objective of these experiments was to evaluate the effects of applying wood-sourced biochar to the feedlot pen surface during winter and summer feeding periods on manure nutrient capture of N and P.

## MATERIALS AND METHODS

All procedures and animal management practices were approved by the University of Nebraska-Lincoln Institutional Animal Care and Use Committee (IACUC approval # 1785).

### Feedlot Performance

Two experiments were conducted at the University of Nebraska-Lincoln (UNL) Eastern Nebraska Research, Extension and Education Center (ENREEC) near Mead, NE, to evaluate the impact of biochar addition to the feedlot pen surface on manure nutrient capture. A 186-d feedlot finishing experiment was conducted from December to June (WINTER) and a subsequent 153-d feedlot finishing experiment was conducted from June to November (SUMMER) of 2020.

**Figure 1.**
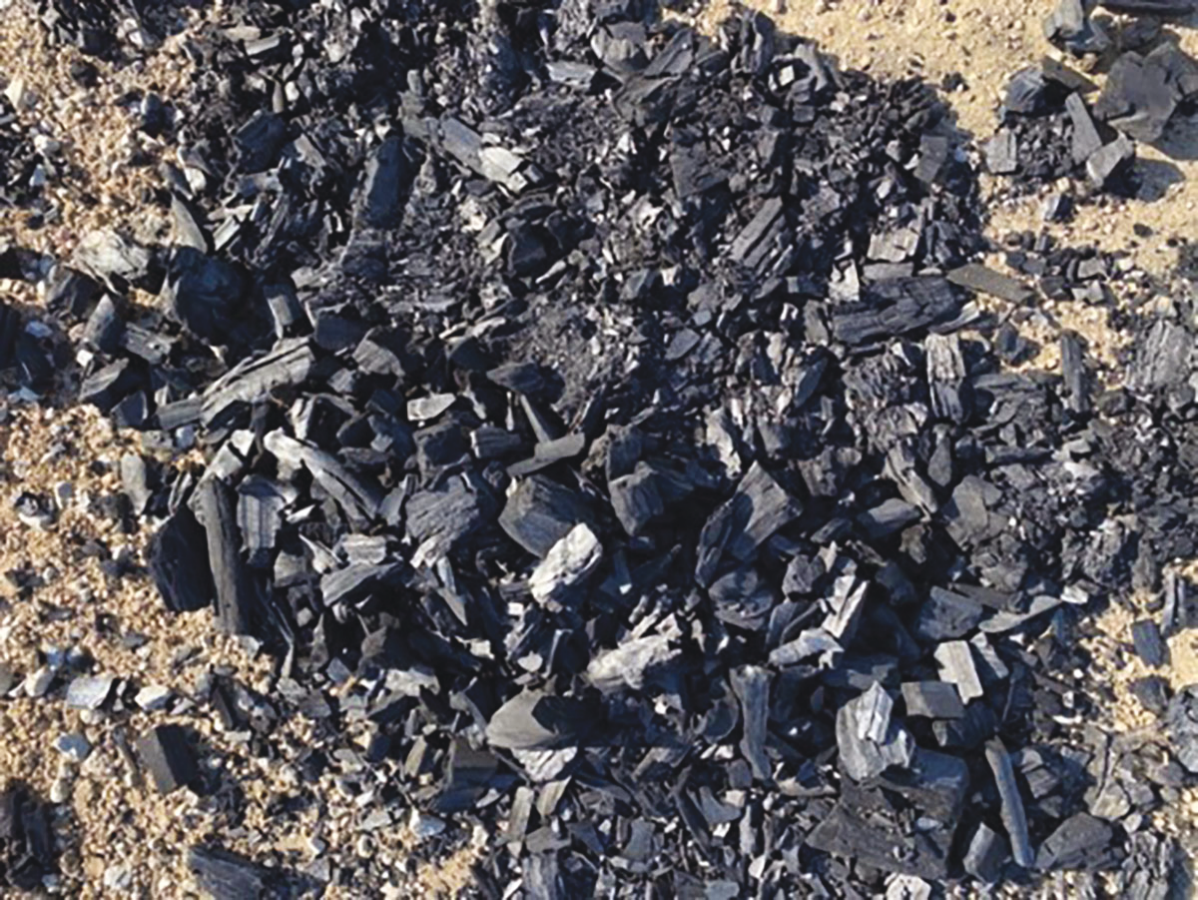
Biochar as applied to the feedlot pen surface (WINTER and SUMMER experiments).

**Figure 2.**
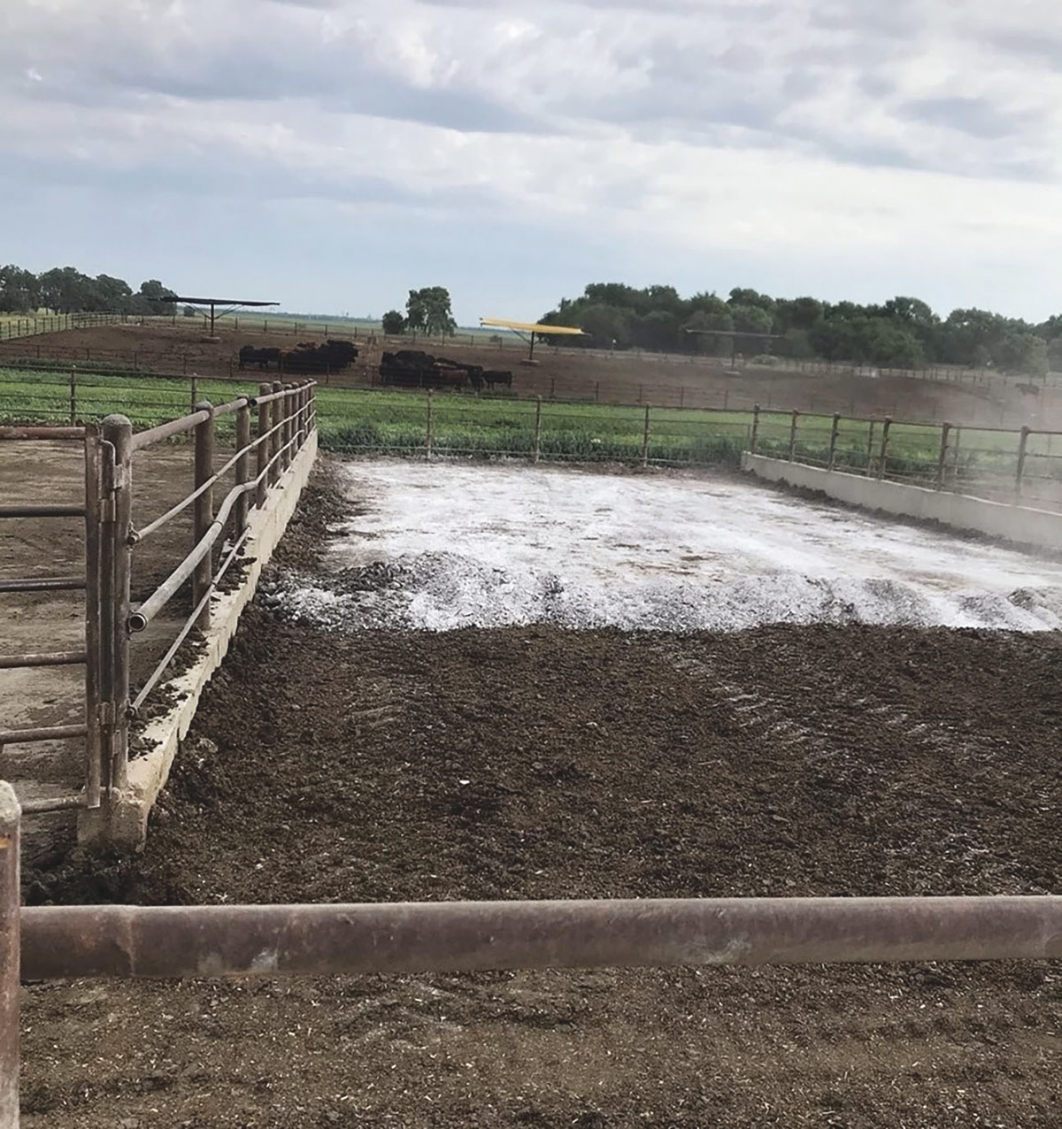
Feedlot pen immediately after application of hydrated lime (WINTER experiment only).

In WINTER, crossbred calves (*n* = 150; initial BW = 274 kg; SD = 7 kg) were assigned to three treatments; negative control, biochar application to pen surface (Figure 1), and hydrated lime (calcium hydroxide) application to pen surface (Figure 2). Unprocessed biochar made from eastern red cedar trees was applied to the pen surface in equal weights [123 kg dry matter (DM) per pen] at experiment initiation in December and again in February (total of 246 kg DM per pen; at a rate of 1.40 kg/m^2^). The weight of unprocessed biochar distributed to each treatment pen was targeting approximately 5% of excreted manure DM over the experimental period. Lime treatment was applied to the pen surface (308 kg DM per pen; at a rate of 1.75 kg/m^2^) one day prior to shipping cattle for harvest. Biochar and lime treatments were applied to the soil-surface area of the pen, with no application to the concrete apron. The application of lime to the feedlot pen surface was in cooperation with UNL Civil and Environmental Engineering to determine the impact of lime on microbial activity on the pen surface, as a means to reduce pathogenic bacteria load on the hide of the animal at harvest. The alkaline stabilization properties of lime are hypothesized to reduce antimicrobial-resistant bacteria in cattle manure by raising the pH level of the pen surface, thus making conditions unfavorable for the growth of pathogenic bacteria (Avery et al., 2009). However, increasing the pH of the pen surface may increase NH_3_ volatilization, as the speed of hydrolysis from NH_4_ to NH_3_ is increased as pH of the pen surface approaches an equilibrium point at pKa greater than 9.

Pens were assigned randomly to treatments (5 pens/treatment) and steers were assigned randomly to pens (10 steers/pen). The WINTER finishing diet contained 51% high-moisture corn (HMC), 20% Sweet Bran (Cargill Corn Milling, Blair, NE), 15% corn silage, and 10% modified distillers grains, with a mean dietary crude protein (CP) concentration of 13.7% and dietary P concentration of 0.45% (Table 1).

**Table 1.** Composition of diet (DM) fed to steers in WINTER and SUMMER mass balance experiments

Item	Experiment	
	WINTER	SUMMER
Ingredient, % dietary DM		
High-moisture corn	51	51
Sweet bran^1^	20	40
Corn silage	15	–
MDGS^2^	10	–
Cornstalks	–	5
Supplement^3^		
Finely ground corn	1.89	1.89
Limestone	1.63	1.63
Salt	0.300	0.300
Tallow	0.100	0.100
Beef trace mineral	0.050	0.050
Rumensin-90^4^	0.015	0.015
Vitamin A-D-E	0.014	0.014
Tylan-40^5^	0.010	0.010
Nutrient analysis, %^6^		
Dry matter	57.7	66.9
Organic matter	92.6	92.4
Crude protein	13.7	14.5
Neutral detergent fiber	18.89	20.07
P	0.45	0.53
K	0.69	0.73
S	0.20	0.21

Sweet bran = branded wet corn gluten feed produced by Cargill (Cargill Corn Milling, Blair, NE).

MDGS = modified distillers grains plus solubles.

Supplement fed at 4% of dietary DM. Ractopamine hydrochloride (Optaflexx; Elanco Animal Health; Indianapolis, IN) was fed for last 28 d prior to harvest in WINTER experiment targeted to provide 300 mg/steer daily and replaced finely ground corn in the supplement. Trace mineral premix contained 6% Zn, 5% Fe, 4% Mn, 2% Cu, 0.3% Mg, 0.2% I, and 0.05% Co. Vitamin premix contained 30,000 IU of Vitamin A, 6,000 IU of Vitamin D, and 7.5 IU of Vitamin E per gram.

Monensin (Rumensin; Elanco Animal Health) targeted to provide 33 mg/kg dietary DM.

Tylosin (Tylan; Elanco Animal Health) targeted to provide 90 mg/steer daily.

Nutrient composition calculated from nutrient analysis of individual ingredients.

In SUMMER, crossbred yearlings (*n* = 80; initial BW = 339 kg; SD = 7 kg) were assigned to two treatments; negative control and biochar application to pen surface. Unprocessed biochar was applied to the pen surface in equal volumes (123 kg DM per pen) at experiment initiation in June and again in August (total of 246 kg DM per pen; at a rate of 1.40 kg/m^2^). The same pens were used for both the WINTER and SUMMER experiments, with treatments maintained on the same pens (5 pens/treatment). Steers were assigned randomly to pens (8 steers/pen). With fewer steers per pen and fewer days on feed in SUMMER compared to WINTER, biochar application targeted approximately 10% of excreted manure DM for the SUMMER experiment. The SUMMER finishing diet contained 51% HMC, 40% Sweet Bran, and 5% cornstalks, with a mean dietary CP concentration of 14.5% and dietary P concentration of 0.53% (Table 1).

Prior to initiating each of the experiments (WINTER and SUMMER), all steers were individually identified and processed upon arrival at the ENREEC feedlot. Steers were administered a modified live vaccine for the prevention of infectious bovine rhinotracheitis, bovine viral diarrhea, parainfluenza 3, bovine respiratory syncytial virus, Mannheimia haemolytica, and Pasteurella multocida (Vista, Merck Animal Health, Summit, NJ), a killed vaccine for Clostridial toxoids and *Histophilus somni* (Ultrabac 7/Somubac, Zoetis Inc, Kalamazoo, MI), and an injectable solution for the treatment and control of gastrointestinal roundworms, lungworms, eyeworms, lice, and mites (Dectomax, Zoetis Inc.). Steers were limit-fed a common diet of 50% alfalfa hay and 50% Sweet Bran offered at 2% of BW for five days to equalize gut fill (Watson et al., 2013). Steers were weighed in the morning before feeding on days 0 and 1 of the experiments and weights were averaged to establish initial BW. Feed was delivered to pens once daily at approximately 0800 h, aiming for trace amounts of feed in the bunk during the time of feeding. Weekly grab samples of dietary ingredients were collected for determination of DM and as-fed proportions of ration ingredients were adjusted weekly as required. Weekly feed samples were composited by month and composites were sent to Ward Laboratories LLC (Kearney, NE) for nutritional and chemical analysis. Samples were dried for 48 h at 60 °C in a forced-air oven to determine DM. Organic matter content was determined by ashing samples at 600 °C for 6 h. Total N was determined using a combustion method N analyzer (AOCAC International, 1999: method 4.2.04). Ashing and digestion (AOAC, 1990: method 648.08) of samples, followed by colorimetric analysis using the molybdovanadate method (AOAC, 1990: method 965.17) on a spectrophotometer (Molecular Devices SpectraMAX 250; 400 nm) was used to determine total P content. Neutral detergent fiber content was measured using an Ankom 200 Fiber Analyzer (ANKOM Technology, Fairport, NY).

Steers were implanted with 80 mg trenbolone acetate and 16 mg estradiol (Revalor-IS; Merck Animal Health) on day 1 of the experiments and reimplanted with 200 mg trenbolone acetate and 20 mg estradiol (Revalor-200; Merck Animal Health) on days 76 and 68 for WINTER and SUMMER, respectively. Steers were harvested at a commercial abattoir (Greater Omaha, Omaha, NE) upon completion of the WINTER and SUMMER feeding periods. On the day of shipping, cattle were offered 50% of the previous day’s feed at the regular time of feeding. Cattle were loaded and shipped to the abattoir in the afternoon for slaughter the next morning. Hot carcass weights (HCW) were recorded on the day of slaughter and USDA marbling scores, 12th rib fat thickness, and longissimus muscle (LM) area were recorded after a 48- and 72-h chill for WINTER and SUMMER, respectively. Carcass chill for SUMMER included an additional 24-h compared to WINTER because of plant shutdown during the (USA) Thanksgiving holiday. Calculated yield grade was determined using the following equation (USDA, 2016): 2.50 + (0.98425 × 12th rib fat, cm) + (0.2 × KPH, %) + 0.00837 × HCW, kg) – (0.0496 × LM area, cm^2^), where KPH fat was assumed to average 2.5%. Backfat (12th rib fat) was determined through camera grading. Performance traits including final body weight (BW), average daily gain (ADG), and Gain:Feed (G:F) were calculated based on HCW adjusted to a common dressing percentage of 63.

### Nutrient Mass Balance

Biochar utilized for the WINTER and SUMMER experiments was provided by Sawle Mill (Springview, NE), and was sourced from Eastern red cedar trees. Dry matter of the biochar fluctuated with moisture in the air from 85% to 95% DM with an average of 90% and OM content of 95%. Biochar samples were collected on both application dates for WINTER and SUMMER experiments and sent to Control Laboratories (Watsonville, CA) for chemical analysis. On a DM basis, carbon (C) content of the biochar was 80.3%, with a surface area of 233 m^2^/g, bulk density of 155.4 kg/m^3^, total N content of 0.6% of DM mass, and pH of 6.3. Biochar particle size distribution ranged from 0.5-mm to 50-mm, with approximately 70% of biochar sampled sizing >8-mm. Unprocessed biochar was utilized in both WINTER and SUMMER, expecting that cattle hoof action would reduce particle size.

The nutrient mass balance experiments were conducted similar to experiments described by Bierman et al. (1999), Erickson and Klopfenstein (2001a), and Luebbe et al. (2012) on open dirt feedlot pens. Pens utilized in both WINTER and SUMMER experiments had a soil-based surface area of 176 m^2^ and total pen surface area of 262 m^2^, with a pen slope of 3%, and feed bunk space of 17.6 and 22.0 cm per steer in WINTER and SUMMER, respectively. Each pen was separated from adjacent pens by a concrete curb in the shared fence line.

Twelve soil core samples (15-cm depth) were taken from each pen at the start (before cattle entered pens) and end of each experiment to correct for any change in soil nutrient concentration and to determine pen cleaning equivalence. Soil cores were collected by dividing the pens into 12 grids and collecting one core sample per grid to represent pen average. Before experiment initiation, the concentration of nutrients in soil cores averaged 3.5% OM, 0.10% P, and 0.21% N, all on a DM basis. Once cattle were removed from pens on day 186 (WINTER) and 153 (SUMMER) for slaughter, the pen surfaces were cleaned (<24 h) in replication across treatments with a box scraper to remove waste material with minimal soil removal, and a skid steer to scrape the concrete apron and pile manure. The manure pile was mixed using the skid steer and during load out from the concrete apron two separate sets of manure samples were collected for nutrient analysis (*n* = 20 samples per pen; approximately 100 g each) and DM determination (*n* = 10). Manure subsamples were composited by pen, resulting in two composites per pen for manure. Trucks were weighed to determine the weight of material removed from each individual pen before transferring it to a storage lot.

All manure samples were frozen at −4 °C to conserve N until either oven-dried for DM analysis or freeze-dried for nutrient analysis. Manure samples (analyzed in duplicate) were oven-dried at 60 °C for 48 h (AOAC International, 1999; method 4.2.03) to determine DM content and, subsequently, DM removal from each pen. Pen soil core samples and manure samples collected for nutrient analysis were freeze-dried, ground to pass through a 1-mm screen (Wiley Mill), composited by pen (two composites per pen for manure), and sent to a commercial laboratory (Ward Laboratories LLC, Kearney, NE) for analysis of N and P content (outlined by Homolka et al., 2021) utilizing the same procedures described for diet ingredients.

### Statistical Analysis

Cattle performance, carcass characteristics, and nutrient mass balance data were analyzed using the MIXED procedure of SAS (SAS Institute, Inc., Cary, NC) with the pen as the experimental unit for both WINTER and SUMMER experiments.

Nutrient mass balance data were calculated using the methods outlined by Luebbe et al. (2012) and Homolka et al. (2021). Nutrient intake was determined based upon monthly feed ingredient composites and feed delivery and refusals on a pen basis. Nutrient retention and excretion were calculated utilizing methods established by NASEM (2016) and ASABE (2014). The N and P retained by the animal were calculated utilizing energy, protein, and P retention equations (NASEM, 2016). Nutrient excretion was then calculated by subtracting nutrient retention from nutrient intake. Runoff was not measured in this experiment because at our facility runoff losses generally account for only 1.5% to 2.5% of fed N and 4% to 6% of fed P from open lot pens. Total nutrient loss (kg/steer) was calculated by subtracting recovered manure nutrients (corrected for soil cores) from excreted nutrients. Significance was considered at *α* ≤ 0.05 and a tendency was considered at 0.05 < *α* ≤ 0.10.

## RESULTS AND DISCUSSION

### Cattle Performance

There were no significant differences in dry matter intake (DMI; *P* = 0.10), average daily gain (ADG; *P* = 0.50) or Gain:Feed (*P* = 0.98) due to pen treatment in WINTER (Table 2). Carcass characteristics were not impacted by pen treatments for cattle in WINTER (*P* ≥ 0.50). There was a significant increase in carcass-adjusted final BW (*P* = 0.05) and ADG (*P* = 0.05) for steers in biochar-amended pens in SUMMER (Table 3) compared to control, and no difference between treatments for DMI (*P* = 0.48). This increase in gain tended to improve feed efficiency (*P* = 0.08) for steers in biochar-treated pens compared to control and resulted in significantly heavier HCW (*P* = 0.05) for biochar treatment. Results from SUMMER showed no difference in other USDA carcass parameters, including LM area, marbling, 12th rib fat, or yield grade (*P* ≥ 0.76).

**Table 2.** Performance and carcass characteristics for steers fed the same diet with different pen amendments in WINTER phase

	Treatments^1^			SEM	*P*-value
	Control	Biochar	Lime		
Performance					
Initial BW, kg	274	274	274	1.3	0.95
Final BW^2^, kg	618	622	628	5.7	0.50
DMI, kg/d	10.0	10.1	10.3	0.04	0.10
ADG, kg	1.86	1.87	1.90	0.029	0.50
Gain:Feed	0.185	0.186	0.185	0.0022	0.98
Carcass characteristics					
HCW, kg	390	392	396	3.5	0.50
LM area, cm^2^	86.5	87.7	87.7	1.29	0.76
Marbling^3^	472	463	476	13.71	0.79
12^th^ rib fat^4^, cm	1.45	1.40	1.42	0.051	0.79
Calculated yield grade	3.43	3.38	3.40	0.050	0.78

Control = no treatment applied; Biochar = eastern red cedar biochar applied to the soil surface of the pen at 1.40 kg dry matter/m^2^; Lime = applied 1 d prior to cattle harvest at 1.75 kg dry matter/m^2^.

Carcass adjusted final BW determined from HCW divided by common dressing percentage of 63%.

Marbling score: 400= small^00,^minimum required for U.S. Low Choice.

12th rib fat, cm determined using camera grading.

**Table 3.** Performance and carcass characteristics for steers fed the same diet with different pen amendments in SUMMER phase

	Treatments^1^		SEM	*P*-value
	Control	Biochar		
Performance				
Initial BW, kg	339	339	1.08	0.92
Final BW^2^, kg	665	682	5.99	0.05
DMI, kg/d	12.1	12.2	0.045	0.48
ADG, kg	2.13	2.24	0.039	0.05
Gain:Feed	0.176	0.184	0.0032	0.08
Carcass characteristics				
HCW, kg	419	429	5.3	0.05
LM area, cm^2^	92.7	93.0	1.67	0.89
Marbling^3^	492	499	15.1	0.76
12th rib fat^4^, cm	1.50	1.50	0.090	0.98
Calculated yield grade	3.48	3.48	0.065	0.98

Control = no treatment applied; biochar = eastern red cedar biochar applied to the soil surface of the pen at 1.40 kg dry matter/m^2^.

Carcass adjusted final BW determined from HCW divided by common dressing percentage of 63%.

Marbling score: 400= small^00^, minimum required for U.S. Low Choice.

12th rib fat, cm determined using camera grading.

The significant increase in ADG and final BW for SUMMER steers on biochar-amended pens may have been influenced by the moisture content of the pen surface (Maharjan and Wilke, 2021); however, pen surface moisture across time was not measured in this experiment. Maharjan and Wilke (2021) spread coal char (30% C) sourced from a sugar beet factory on feedlot pens in Nebraska at a rate of 568 kg per steer, which was 20 times the rate spread in the present experiment. Results from soil moisture sensors fixed on the pen surface indicated that following a series of snowfall events, the char amended pens were significantly drier than the controls. The biological and chemical properties of wood-sourced biochar may absorb water (Zhang, Chen, and You, 2016); thereby reducing the impact of moisture on the pen surface and lessening the negative impacts of mud. The weight of biochar added to the pen surface per steer was greater in the SUMMER (31 kg) compared to WINTER (25 kg) experiment (because of fewer steers per pen in SUMMER), and the SUMMER feeding period had greater precipitation compared to WINTER (Table 4). In addition, biochar in the SUMMER experiment was spread at experiment initiation in June and again in August, with precipitation for the month of July reaching volumes that were over double that of the 25-y average of annual precipitation. These factors suggest that the greater volume of biochar and the timing of application (during higher-than-average precipitation conditions) may have reduced the negative implications that moisture causes on the feedlot pen surface. Muddy feedlot pens contribute to poor animal performance and increased labor for feedyard personnel, ultimately increasing the cost of gain (Mader and Griffin, 2015). The 25-yr average of annual precipitation near Mead, NE, equates to approximately 76 cm per year (HPRCC, 2021; Table 4). Grandin (2016) suggests that controlling mud in open-lot pens becomes increasingly difficult when precipitation is greater than 51 cm per year. On average, steers on the biochar-amended pens in SUMMER were 17 kg heavier in carcass-adjusted final BW compared to controls, suggesting that the addition of biochar to the pen surface indirectly benefited steer performance.

**Table 4.** Monthly precipitation (cm) for WINTER and SUMMER compared to 25-year average monthly precipitation for the Eastern Nebraska Research, Education and Extension Center located near Mead, NE.

Month	Phase		25-tear Average^1^
	WINTER	SUMMER	
December	6.50	-	2.95
January	3.28	-	1.47
February	0.28	-	2.08
March	4.24	-	3.71
April	1.91	-	7.70
May	11.63	-	12.75
June^2^	1.63	6.27	11.63
July	–	14.66	6.81
August	–	3.23	9.83
September	–	4.01	8.05
October	–	0.94	5.79
November	–	3.18	3.18
Total precipitation^3^	29.47	32.29	75.95

Monthly average precipitation (cm) from 1995 to 2020 for Mead, NE, sourced from the High Plains Regional Climate Center (HPRCC, 2021).

WINTER phase included precipitation from June 1st through 18th, SUMMER phase included precipitation from June 19th through 30th.

Total precipitation for WINTER and SUMMER combined = 61.76 cm.

### Nutrient Mass Balance

In the WINTER experiment (Table 5), N intake, retention, and excretion were not different between treatments (*P* ≥ 0.42). The concentration of N in manure tended to differ between treatments (*P* = 0.07), with biochar-amended pens having the greatest manure N as a percent of manure DM. The manure N concentration as a percent of OM was the greatest for the control pens, and lowest for the lime-amended pens, with biochar-amended pens as an intermediate (*P* < 0.01) suggesting the change in manure N concentration on a DM basis is related to soil contamination in as-removed manure from pens. Less soil was removed from biochar-amended pens based on ash/OM concentrations resulting in more N in manure due to less soil removed from the pen surface. The concentration of N as a % of OM being greatest for control pens suggests the change in N concentration as % of manure DM is not due to capturing more N due to biochar. In WINTER, P intake, retention, and excretion were not different between treatments (*P* ≥ 0.38) and there was no difference between treatments in the concentration of manure P (*P* = 0.23) as a percent of manure DM. Manure nutrient losses were not different for all treatments and averaged 54% loss of N (*P* = 0.37) and 0.43% loss of P (*P* = 0.87). The lime treatment had the highest numerical retention of N in the manure (*P* = 0.15), which was not expected based on the relationship between pH and NH_3_ volatilization and the high alkalinity of calcium hydroxide. The pH of the pen surface influences the speed of NH_3_ volatilization, where the ideal pH conditions for rapid volatilization are neutral (pH 7) to basic (pH 10; Hartung and Phillips et al., 1994). The pKa of NH_3_ is approximately 9.2; therefore, when the surface pH drops below 6.5, essentially all of the NH_3_ is in the non-volatile NH_4_ form rather than the volatile NH_3_ form (Rhoades et al., 2010). The soil surface of lime treatment pens increased to a pH of 12.5 after lime application, with pH remaining above 10 for 72 h after application. During this same period, the pH of the soil surface in control pens averaged 8.9 (Mware et al., 2022).

**Table 5.** Effect of biochar and lime pen amendments on manure nitrogen (N), phosphorus (P) and organic matter (OM) during WINTER^1^

	Treatments^2^		Lime	SEM	*P*-value
	Control	Biochar			
Nitrogen					
N intake, kg/steer	41.4	41.8	42.4	0.5	0.46
N retention^3^, kg/steer	7.3	7.4	7.4	0.1	0.60
N excretion^4^, kg/steer	34.1	34.4	34.9	0.5	0.42
N manure^5^, kg/steer	15.4	15.2	16.8	0.6	0.15
N lost^6^, kg/steer	18.7	19.2	18.1	0.9	0.68
N Lost^7^, %	54.9	55.7	51.7	2.0	0.37
Phosphorus					
P intake, kg/steer	8.5	8.6	8.7	0.1	0.43
P retention^3^, kg/steer	1.8	1.8	1.8	0.01	0.60
P excretion^4^, kg/steer	6.7	6.8	6.9	0.1	0.38
Manure P^5^, kg/steer	6.7	6.6	7.0	0.4	0.75
P lost^6^, kg/steer	0.005	0.181	-0.086	0.363	0.87
P lost^7^, %	-0.1	2.7	-1.3	5.3	0.87
Manure characteristics					
DM, %	92.0	91.1	91.7	0.01	0.42
DM removed, kg/steer	446.7	360.1	450.8	35.6	0.17
OM, % of DM	35.1	40.3	37.8	1.5	0.09
OM removed, kg/steer	157.8	144.7	168.7	13.7	0.48
Manure N, % of DM	1.57	1.71	1.51	0.06	0.07
Manure N, % of OM	4.50^a^	4.25^b^	4.00^c^	0.07	<0.01
Manure P, % of DM	0.69	0.76	0.69	0.031	0.23
Ash, % of DM	64.9	59.7	62.2	1.5	0.09

Values expressed as kg/steer over the entire feeding period (186 days on feed).

Control = no treatment applied; Biochar =eastern red cedar biochar applied in December and February at 123 kg per pen for each application; Lime = applied 1 d prior to cattle harvest approximately 308 kg per pen.

Calculated using the NASEM (2016) net energy, protein, and P retention equations.

Calculated as nutrient intake—retention.

Manure N or P with correction for soil N or P. Soil nutrient concentration evaluated before and after WINTER experiment to account for all nutrients remaining or in excess on the pen surface.

Calculated as nutrient excretion (N or P)—nutrient in manure (N or P).

Calculated as nutrient (N or P) lost divided by nutrient (N or P) excretion.

Conversely, it has been well established in the literature that the warming effect of solar radiation on the pen surface depends on the soil color, in which dark-colored soil surfaces absorb more energy than light-colored soil surfaces, and light-colored soil surfaces reflect more solar radiation (Harpstead et al., 2001). The hydrated lime treatment created a white layer of residue on the pen surface, which may have reduced the pen surface absorption of radiant energy resulting in a cooler surface and thereby reducing NH_3_ volatilization. However, the lime treatment was only applied to the pen surface on the final day of the experiment which limited impacts.

The quantity of DM removed from the pen surface in WINTER was similar for all treatments (*P* = 0.17) and may have been influenced by the abnormally dry pen conditions at the conclusion of the WINTER experiment. Oven-dried manure samples averaged 92%, 91%, and 92% DM content for control, biochar, and lime, respectively, which was drier than expected for WINTER feeding periods, averaging around 64% in previous literature (Homolka et al., 2021).

In the SUMMER experiment (Table 6), N intake and excretion were not different between treatments (*P* ≥ 0.35) and steers in biochar-amended pens had significantly greater N retention compared to the control (*P* = 0.04). The intake and excretion of P were not different between treatments (*P* ≥ 0.35), and P retention was significantly greater for the biochar treatment compared to control (*P* = 0.03). Steers fed in biochar-treated pens had significantly greater ADG (*P* = 0.05), and final BW (*P* = 0.05), resulting in greater calculated N and P retention compared to control steers. Manure N concentration as a percent of manure DM tended to be greatest for biochar treatment (*P* = 0.07) with no difference in manure P concentration as a percent of manure DM (*P* = 0.36). The increase in manure N as a % of DM was a reflection of removing more OM (originating feces and urine from cattle) and less ash (soil). The manure N concentration as a percent of OM tended to be greater for the control treatment (*P* = 0.09). Manure nutrient losses were not different for biochar and control pens with 71% of excreted N (26.3 kg/steer; *P* ≥ 0.79) and 10% of excreted P (0.85 kg/steer; *P* = 0.88) lost during the SUMMER experiment. Loss of 71% of excreted N is consistent with a 15-study analysis from 1999 to 2015 measured at the same location as these experiments, reporting an average of 73% loss of excreted N in SUMMER feeding periods (Homolka et al., 2021).

**Table 6.** Effect of biochar as a pen amendment on manure nitrogen (N), phosphorus (P) and organic matter (OM) during SUMMER^1^

	Treatments^2^		SEM	*P*-value
	Control	Biochar		
Nitrogen				
N Intake, kg/steer	43.4	43.9	0.6	0.35
N retention^3^, kg/steer	6.5	6.8	0.1	0.04
N excretion^4^, kg/steer	36.9	37.1	0.5	0.67
N manure^5^, kg/steer	10.5	11.2	1.8	0.78
N lost^6^, kg/steer	26.4	25.9	2.0	0.85
N lost^7^, %	71.6	69.6	5.0	0.79
Phosphorus				
P intake, kg/steer	9.8	9.9	0.1	0.35
P retention^3^, kg/steer	1.6	1.7	0.01	0.03
P excretion^4^, kg/steer	8.2	8.3	0.1	0.69
Manure P^5^, kg/steer	7.5	7.3	1.0	0.90
P lost^6^, kg/steer	0.7	1.0	1.1	0.88
P lost^7^, %	8.5	11.4	13.1	0.88
Manure characteristics				
DM, %	55.2	55.8	0.01	0.72
DM Removed, kg/steer	267.1	233.6	16.6	0.08
OM, % of DM	44.2	50.1	1.94	0.06
OM removed, kg/steer	117.0	116.1	5.2	0.87
Manure N, % of DM	2.01	2.20	0.06	0.07
Manure N, % of OM	4.57	4.39	0.07	0.09
Manure P, % of DM	1.06	1.13	0.06	0.36
Ash, % of DM	55.8	49.9	1.94	0.06

Values expressed as kg/steer over entire feeding period (153 days on feed).

Control = no treatment applied; Biochar =eastern red cedar biochar applied in June and August at 123 kg per pen for each application.

Calculated using the NASEM (2016) net energy, protein, and P retention equations.

Calculated as nutrient intake—retention.

Manure N or P with correction for soil N or P. Soil nutrient concentration evaluated before and after SUMMER experiment to account for all nutrients remaining or in excess on the pen surface.

Calculated as nutrient excretion (N or P)—nutrient in manure (N or P).

Calculated as nutrient (N or P) lost divided by nutrient (N or P) excretion.

Oven-dried manure samples averaged 55% and 56% DM content for control and biochar treatments, respectively, suggesting that the feedlot pen surfaces in SUMMER were considerably wetter than the 15-study average of 70% DM reported by Homolka et al. (2021). The quantity of manure DM removed from the pen surface in SUMMER tended to be less for the biochar amended treatment (*P* = 0.08). The concentration of ash in the removed manure tended to be less for the biochar-amended pens compared to the control (*P* = 0.06), indicating that less soil was removed from the pen surface in biochar-amended pens. Due to the wet conditions during feedlot pen cleaning, the manure–soil interface may have been difficult for the equipment operator to identify. Differences in ash content could also be attributed to differences in C losses as CO_2_ or CH_4_ from the pen surface.

It was hypothesized that biochar addition to the feedlot pen surface would increase manure N capture because of the high C content (80.3% C on DM basis) of the biochar utilized in this experiment. In both WINTER and SUMMER, biochar addition to the pen surface tended to increase manure N as a percent of manure DM (*P* = 0.07) and manure OM content (*P* ≤ 0.09), but this increase in N concentration did not translate into greater kg of N or P removed from the biochar-amended pens, because there was less manure DM removed from these pens. Results from these experiments were incongruent with previous literature regarding various feedlot management strategies aiming to increase the C content of the pen surface to reduce nutrient loss. Lory et al. (2002) looked at the impact of sawdust application to the feedlot pen surface on N losses in winter and summer feeding phases, concluding that application of a product high in C to the pen surface (such as sawdust) reduced N losses during summer months. Adams et al. (2004) utilized sawdust as an OM addition to the pen surface, comparing it to a treatment where cattle had a dietary inclusion of 30% greater corn bran in the diet, and a negative control treatment (no sawdust or dietary bran addition). The higher inclusion of bran in the diet was designed to decrease diet digestibility, thereby increasing OM excretion onto the pen surface. Adams et al. (2004) found that adding OM to the pen surface, either as a pen amendment (sawdust) or by increasing OM excretion, increased manure N content by 20% compared to the control during the winter/spring months. Bierman et al. (1999) and Erickson and Klopfenstein (2001b) reported similar results to Adams et al. (2004), where feeding less-digestible diets resulted in greater OM excretion on the pen surface and increased manure nutrient capture.

Manure N loss for the WINTER experiment averaged 54% of excreted N and manure N loss for the summer experiment averaged 71% across treatments. These observations are consistent with Homolka et al. (2021) who reported losses of 50% and 73% of excreted N for winter and summer feeding periods, respectively. A greater N loss observed in summer feeding experiments is well defined in the literature, where warmer temperatures (> 25 °C) increase the speed of urea hydrolysis, equating to faster rates of NH_3_ volatilization (Moyo, Kissel, and Cabrera, 1989). Kissinger et al. (2007) summarized the manure characteristics of 15 open-lot pens of cattle (*n* = 6,366) sourced from Nebraska feedlots over the course of a 1-yr feeding period, reporting N losses from volatilization and runoff to be 53% and 67% of fed N for winter and summer, respectively. In a Texas feedlot experiment conducted by Todd et al. (2008), N loss from manure via NH_3_ volatilization was 68% in the summer months and 36% in the winter months. In the present experiment, the quantity of manure P collected in SUMMER, 48 g/steer daily, and WINTER, 36 g/steer daily, were substantially greater than values reported by Homolka et al. (2021), who observed 17 g/steer daily in summer and 28 g/steer daily in winter. Kissinger et al. (2007) reported an average of 26 and 37 g/steer daily in summer and winter feeding periods, respectively, from 6 commercial feedlots. Of the 15 experiments that Homolka et al. (2021) reported, the average dietary P concentration was 0.36%, with the maximum dietary P concentration at 0.49% of dietary DM. The diets fed in SUMMER and WINTER had a P concentration of 0.53% and 0.45% of dietary DM, respectively, influenced by the greater inclusion of byproduct in the diet compared to Homolka et al. (2021).

On average, the quantity of manure DM removed was 27% greater and the quantity of manure N removed was 28.5% greater in the WINTER experiment than in the SUMMER experiment. These results are consistent with Kissinger et al. (2007), who summarized data from six Nebraska feedlots representing 15 feeding pens with 40 separate lots of cattle fed in those pens, equating to 6,366 head of cattle; concluding that manure harvested after a winter feeding period is about 20% more than that of manure harvested following a summer feeding period, and that manure harvested after winter feeding has significantly greater retention of excreted N compared to summer. The significant increase in N recovery in manure during winter is likely due to less N volatilization during winter feeding months compared to summer feeding months (Todd et al., 2008).

Results from these experiments suggest that the addition of unprocessed eastern red cedar biochar to the feedlot pen surface (1.40 kg/m^2^) did not increase manure nutrient retention and did not reduce N losses. In both experiments, biochar addition to the pen surface tended to increase manure N as a percent of manure DM, but this increase in N concentration did not translate into greater kg of N or P removed from the feedlot pens. Biochar addition to the feedlot pen surface did improve the growth performance of steers in the SUMMER feeding experiment, although no differences were found in growth performance for the WINTER feeding experiment.
